# National Board of Health

**Published:** 1880-02

**Authors:** 


### NATIONAL BOARD OF HEALTH.

We have before alluded to this organization as one established for the express purpose of carrying out certain quarantine projects. This was evident from the very inception of the enterprise, and our opposition was expressed at the beginning. The question whether quarantine be necessary or at all useful was not so much pressed as that of the best means of enforcing it inland and coastwise. The question of its benefit should have been first satisfactorily proven before the commerce of the country and the convenience and well being of the people should be disturbed by it; for, as expected by the opponents of quarantine, we have found persistent determination on the part of the board to enforce it to prevent yellow fever, after the positive proof of its utter uselessness in Memphis last summer. With money in hand to accomplish a certain purpose, it is not likely those who handle it will be convinced that it should be given up for anything else.

We are in receipt of a letter from Dr. J. C. LeHardy, of Savannah, calling our attention to the movement on the part of the official medical quarantinists to secure additional national legislation, to give the board more perfect power to enforce quarantine against the wish of State and municipal authorities. He suggests that we organize opposition to the granting by the Federal Government of this additional power.

Our answer is that we are opposed to the whole thing. The National Board of Health and the National Sanitary Association were "conceived in sin and brough forth in iniquity." They cannot be changed from their preconceived and determined purpose otherwise than by abolishing the whole concern, or by withholding appropriations which amounts to the same with those who handle the money. The question, "Is quarantine necessary to prevent yellow fever?" should first be settled.

There is a tendency in this country to medical, civil and military centralization, which, like Dr. LeHardy, we oppose, and on this ground if no other, would speak in opposition to the proposed additional power sought by the quarantinists.

